# Development and assessment of a self-management intervention for urinary incontinence among patients with prostate cancer: protocol for a randomized feasibility study

**DOI:** 10.1186/s12894-023-01367-7

**Published:** 2023-11-18

**Authors:** Ching Hui Chien, Kuan Lin Liu, Chun Te Wu, Cheng Keng Chuang, Kai Jie Yu, Po Hung Lin, Xuan Yi Huang, See Tong Pang

**Affiliations:** 1 School of Nursing, National Taipei University of Nursing and Health Sciences, No. 365, Ming-te Road, Peitou District, Taipei City, 112 Taiwan; 2 Division of Urology, Department of Surgery, Chang Gung Memorial Hospital at Keelung, Keelung City, Taiwan; 3 School of Medicine, College of Medicine, Chang Gung University, Taoyuan City, Taiwan; 4 Division of Urology, Department of Surgery, Chang Gung Memorial Hospital at Linkou, Taoyuan City, Taiwan; 5 Graduate Institute of Clinical Medical Sciences, College of Medicine, Chang Gung University, Taoyuan City, Taiwan

**Keywords:** Prostate cancer, Self-management, Social participation, Demoralization, Resilience, Urinary incontinence

## Abstract

**Background:**

Urinary incontinence is a common complication among patients with prostate cancer who have undergone radical prostatectomy. Guided by social cognitive theory and a framework for the recovery of health and well-being, we propose to develop and test a self-management intervention for patients with prostate cancer who experience urinary incontinence after undergoing radical prostatectomy.

**Methods:**

In this study, a self-management intervention for urinary incontinence (SMI-UI) is developed, comprising a mobile self-management application, a self-management handbook, and professional support. The feasibility, acceptability, and effectiveness of this intervention will be assessed. Patient data from the urology departments of two hospitals will be collected through convenience sampling by adopting an experimental, parallel, and random assignment research design. Patients experiencing urinary incontinence after undergoing radical prostatectomy will be invited to participate. After completing the pretest questionnaire, patients will be randomly divided into the experimental and attention control groups. The experimental group will undergo a 12-week SMI-UI, whereas the attention control group will receive an intervention consisting of a single dietetic education information package. The two groups will be tested 12 and 16 weeks after the pretest. In this study, we recorded the sociodemographic and clinical variables; recruitment rate; retention rate; satisfaction with the intervention; cancer-related self-efficacy; urination symptoms and disturbance; social participation and satisfaction; resilience; and demoralization.

**Trial Registration:**

ClinicalTrials.gov ID: NCT05335967 [date of registration 04-04-2022].

## Introduction

Prostate cancer occurs in men, particularly at an older age. It ranks first among cancers in men in terms of incidence in 112 countries and is the leading cause of cancer-related death among men in 48 countries [1]. Radical prostatectomy is the typical method used to treat early-stage prostate cancer, and urinary incontinence is a possible complication of this surgery [2]. The primary cause of urinary incontinence is damage to the sphincter. After radical prostatectomy, most patients with prostate cancer experience varying degrees of urinary incontinence, which gradually improves over time [3]. Qualitative research has indicated that patients with prostate cancer feel despair, embarrassment, and discomfort because of urinary incontinence. The condition limits the presence of such patients in public because of anxiety about urinary incontinence and feeling uneasy about lacking urinary control [4]. Urinary leakage decreases the frequency and intensity with which these individuals engage in physical activities [5].

During disease progression, patients with prostate cancer experience anxiety, depression [6], and fear of cancer recurrence [7]. Demoralization is a major factor that predicts the deterioration of quality of life and the increase in the risks of depression and suicide among patients with cancer [8, 9]. The social adjustment of patients with prostate cancer is profoundly adversely affected during the initial 6 months after diagnosis [10]. In addition, treatment complications, such as urinary incontinence, further affect their social adjustment or participation [10, 11], cancer-related self-efficacy, demoralization [12], and resilience [13].

A self-management intervention can improve prostate cancer patients’ urinary symptoms, psychological distress [14], self-confidence in symptom management [15], and self-efficacy [16]. Various applications (apps) have also been used to assist in self-management interventions. Patients have reported that apps can provide support in symptom management [17]. Thus far, no application-assisted self-management intervention for improving the social participation and satisfaction, demoralization, and resilience of patients with prostate cancer with urinary incontinence after radical prostatectomy has been made widely available.

### Application and effectiveness of a self-management intervention

Self-management is the process where patients participate in some activities with the assistance of health-care providers when they are facing health-related issues. The aim is to maintain a healthy lifestyle, assess physical and psychological status, monitor symptoms, and respond to shocks [18]. The majority of e-health-based self-management is conducted through websites, with a few interventions performed using an application. The duration of interventions is typically 4–24 weeks, with 12 weeks in most studies. All relevant studies have assessed the baseline characteristics and outcomes after the intervention at least once. Self-management interventions based on e-health can improve the self-efficacy and cancer-related fatigue of patients with cancer [19]. A systematic review suggested, but could not statistically demonstrate, that self-management interventions may improve the self-efficacy of patients with prostate cancer [16].

Social cognitive theory is most commonly used to guide studies on self-management interventions for patients with prostate cancer [14, 20, 21]. Self-management interventions can be provided to patients with prostate cancer through face-to-face interaction [14, 22, 23], websites [20, 21, 24–26], telephone calls [15], apps [17, 27, 28], and combined strategies [29–31]. Interventions typically last 4–19 weeks [14, 15, 17, 20, 21, 23–27, 29, 32]. Notably, some studies have involved a single time intervention [30]. In most studies, a single-group design [14, 21, 22, 24, 26, 29, 30] or two-group design [15, 20, 23, 25] has been adopted. Some studies have conducted a pretest and posttest after the intervention [14, 15, 20, 21, 23–26, 29], but others have only conducted a posttest after the intervention [17, 22, 27, 28, 30].

In some studies, a 2–3-month self-management intervention delivered through face-to-face interaction was provided to patients with prostate cancer who experienced urinary incontinence or other urinary symptoms. The findings indicated that such an intervention can improve urinary symptoms [14], urinary incontinence [23], and the emotional status [14] of patients. Other studies have shown that a self-management intervention can improve urinary incontinence [21], urinary function [21, 25], emotional disturbance/psychological well-being [21, 24, 31], and self-confidence regarding symptom management [15, 24].

### Theoretical frameworks

Social cognitive theory states that individual behavior results from the continuous interaction between individual cognition, the environment, and behavioral factors [33]. This theory is frequently used in the development of interventions for behavior modification, particularly for improving self-efficacy [33, 34], which is defined as the self-confidence of individuals in their ability to complete a task or control a situation [35, 36]. Individuals’ self-efficacy can be promoted by providing an individual with a successful experience (performance accomplishments), vicarious experience, emotional arousal, or verbal persuasion [35, 37]. Patients with high self-efficacy view adversity as a challenge; they are characterized by low levels of depression and anxiety [36, 37] and high resilience [36]. The effect of self-efficacy on individuals is not limited to stressful situations. It can generate motivation in individuals and can encourage them to set challenging goals, thereby affecting their decisions and behavior.

In the framework for the recovery of health and well-being of patients with cancer, cancer-related self-efficacy is defined as the self-confidence of patients regarding their self-management of cancer and cancer-related symptoms after primary treatment [38, 39]. The diagnosis and treatment of cancer render individuals vulnerable and decrease their self-confidence. Self-confidence is the key factor that enables patients to practice self-management after cancer treatment. The reconstruction of self-confidence helps patients to manage the problems caused by cancer and its treatment, thus improving their health and well-being [39]. An individual’s cancer-related self-efficacy is strongly affected by preexisting factors (basic demographic attributes), personal factors (disease cognition, general self-efficacy), and environmental factors (social support, health services). Improving cancer-related self-efficacy can help individuals to implement self-management and to be more confident in managing their problems, thereby promoting the recovery of their health and well-being [38, 39]. Improving the self-efficacy of an individual through self-management can improve their health behavior and status [18] and well-being [39].

### Specific objectives

**Fig. 1:**
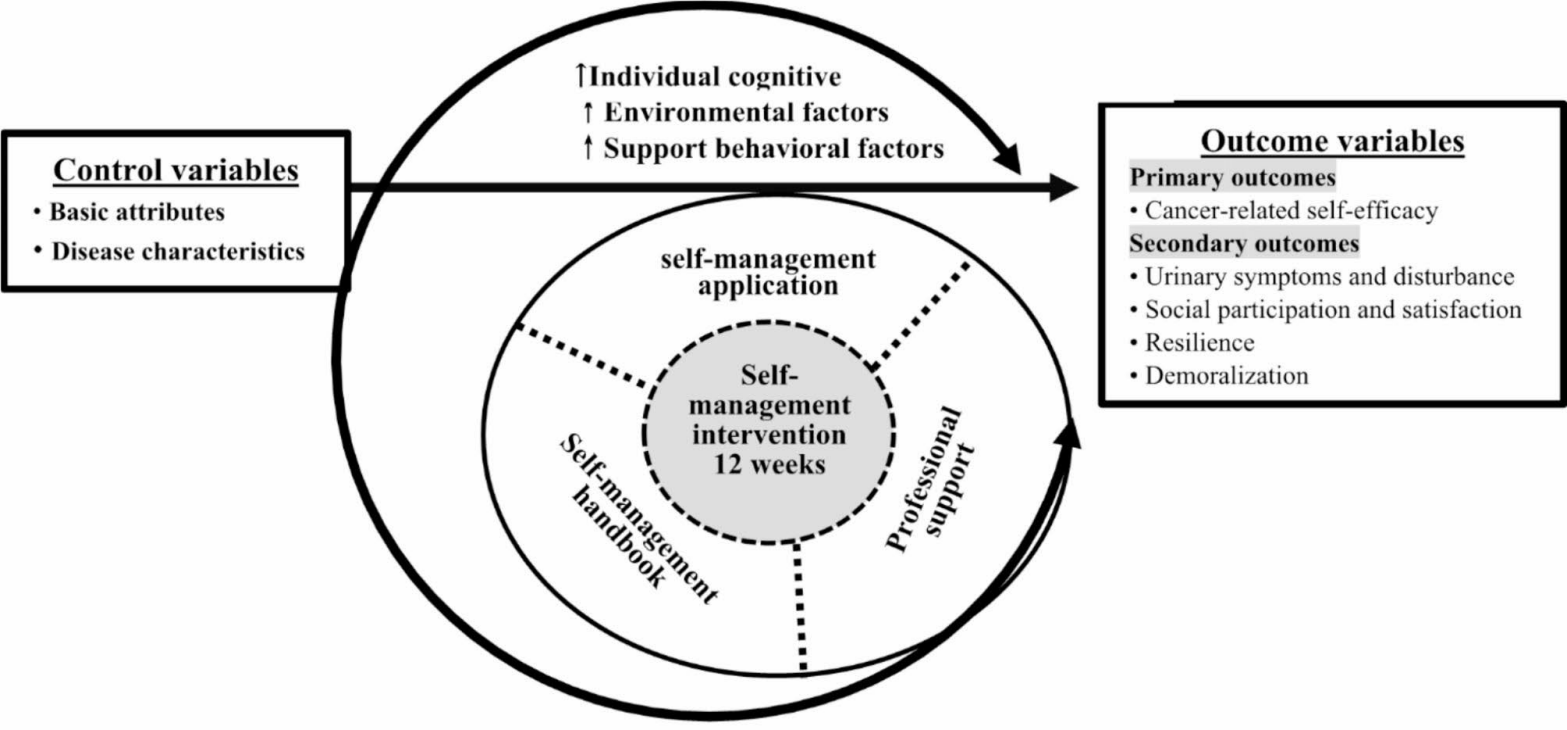
Intervention research framework

Guided by social cognitive theory [33, 35, 37] and the framework for the recovery of health and well-being [38, 39], this study aims to develop a 12-week application-assisted self-management intervention for urinary incontinence (SMI-UI). Moreover, its feasibility, acceptability, and effects on primary outcomes (cancer-related self-efficacy) and secondary outcomes (urinary symptoms and disturbance, social participation and satisfaction, resilience, and demoralization) will be evaluated. The framework is shown in Fig. 1.

## Methods

### Study design and settings

This protocol follows the SPIRIT guidelines [40]. The study will use a parallel random assignment research design with an experimental group and an attention control group [41]. Patients with prostate cancer will be recruited from the urology outpatient department of two hospitals in Taiwan through convenience sampling.

### Eligibility

The inclusion and exclusion criteria are described below. A. Inclusion criteria: (A) Patients with prostate cancer who received radical prostatectomy and suffering from urinary incontinence for at least one week (within two years after surgery). (B) Patients who agree to participate in the research and provide written informed consent. (C) Patients with Eastern Cooperative Oncology Group 0–1 point who can walk independently [42]. (D) Patients who have a smartphone or tablet with a wireless network. (E) Their intimate partner or one of the family members is willing to learn together.

B. Exclusion criteria: (A) A request, with the consideration of family members, that the medical team not tell the patient about the diagnosis or condition of the disease. (B) A history of psychiatric illness, such as dementia, depression, schizophrenia, or bipolar disorder. (C) Suffer from other types of cancer and actively undergoing treatment.

### Discontinuation criteria

After the patients agree to participate in this research, the intervention will be discontinued if they meet any of the following conditions: (1) A physician determines that pelvic floor muscle exercise (PFME) is not suitable for the patient. (2) The patient meets the exclusion criteria. (3) The patient has not experienced urinary incontinence for 1 week or longer prior to the start of the intervention. (4) The patient perceives an improvement in their symptoms of urinary incontinence prior to the commencement of the study. Therefore, although they still experience episodes, they may choose not to participate in the study.

### Research instruments

#### Feasibility

Feasibility is to be assessed based on the recruitment rate and retention rate. The recruitment rate is calculated as follows: [(total number of participants recruited during the study period divided by total number of hospitals)/months of duration of recruitment] [43].

#### Acceptability

Five self-designed questions will be used to determine the acceptability of the developed self-management intervention. The measures include overall satisfaction with the SMI-UI, and the effect and applicability of the intervention in improving self-management ability and self-confidence. The scores for each question range from 0 to 100, with higher scores indicating greater satisfaction.

#### Primary outcome variables

##### Cancer-related self-efficacy

The Chinese version of the self-efficacy scale for patients with cancer [12, 44, 45] will be used to determine the self-efficacy of patients with prostate cancer. It consists of 11 questions, which are scored on a scale of 1–10 points, with a total score of 11–110 points. A higher score denotes better self-efficacy in the self-management of cancer. As per nonparametric item response theory, all 11 items were confirmed to belong to one dimension. The Cronbach’s α is 0.92 for the original English version [38, 45]. The Chinese version has acceptable content validity and internal consistency [12, 44].

#### Secondary outcome variables

##### Urinary symptoms and disturbance

The Chinese version of the Expanded Prostate Cancer Index Composite (EPIC) consists of 50 questions, including several subscales (e.g., urinary, bowel, sexual, and hormonal). In the proposed study, the urinary incontinence and irritant/obstructive bladder symptoms of patients with prostate cancer will be evaluated using the urinary subscale (12 questions) [6, 46]. The score of each item will be transformed to a 0–100 point scale. Higher scores indicate fewer urinary symptoms and disturbances caused by problems with urination. The construct and criterion validity of the English version of the scale have been demonstrated. The Cronbach’s α (internal consistency) of the urinary subscale in the English version is 0.88 [46]. In the Chinese version of the scale, the Cronbach’s α at the urinary subscale is 0.79–0.89 [6]; The Chinese version has been used in relevant studies [6, 47].

##### Resilience

The Chinese version of the 10-question Connor–Davidson Resilience Scale [48–50] will be adopted to measure the resilience of patients with prostate cancer. The scale comprises 10 questions with a 4-point scoring scale (0–3 points). A higher score denotes a higher degree of resilience. The Chinese version was shown to have construct validity, a Cronbach’s α of 0.94, and a 6-month test–retest reliability of 0.66 [50].

##### Demoralization

The demoralization scale (Mandarin version) will be used to measure the demoralization state of patients with prostate cancer [51, 52]. It comprises 24 questions with a 5-point scoring scale (0–4 points). A higher score denotes a higher degree of demoralization [52]. The evidence supports that the scale has criterion-related validity and discriminant validity. The Cronbach’s α value of the total scale is 0.92 [51].

##### Social participation

*A. Chinese version of the social participation scale for elderly adults* The Chinese version of the social participation scale for elderly adults will be used to measure the social participation of patients with prostate cancer. The scale comprises 12 questions in three subscales: leisure sport activities, religious beliefs, and interpersonal relationships. A 5-point scale (1–5 points) will be adopted. A higher score indicates a higher degree of social participation. The scale has construct validity and Cronbach’s α > 0.9 [53].

*B. Social activity participation willingness and satisfaction items* Two self-designed items will be adopted to measure the willingness and satisfaction of patients with prostate cancer to participate in social activities in the past month prior to the evaluation. The questions are “Were you willing to participate in social activities in the last month?” and “Are you satisfied with the number of times you have participated in social activities in the last month?” A Likert scale with a scoring range of 0–10 points will be used. A higher score indicates higher willingness and satisfaction to participate in social activities.

#### Sociodemographic and clinical variables

Basic demographic data to be collected include age, marital status, educational attainment level, religious beliefs, and occupational status. Disease characteristics include cancer stage, last serum concentration of prostate-specific antigen, history of chronic diseases, time since diagnosis (months), and treatment modalities.

#### Screening of individual patients and intervention-assisted questions

##### Question on urinary incontinence experience

A self-designed question on the experience of urinary incontinence will be used to identify patients with prostate cancer who have experienced incontinence in the past week. This question will be used to understand this experience and its severity (0–4 points) in the past week before the evaluation; “0” represents no urinary incontinence experience, whereas “4” indicates urinary leakage during a resting state, such as lying in bed.

##### Question on self-management confidence

Using a previous study as a reference [18], we will design a single question assessment to be administered to the experimental group after each professional support instance. The assessment aims to guage the confidence of individual patients with regards to implementation of related activities in the week following the evaluation. The assessment was based on a 0–10 point scale where “0” represents “not confident at all,” whereas “10” indicates “very confident.”

### SMI-UI

A 12-week self-management intervention for patients with urinary incontinence after radical prostatectomy will be developed based on theoretical frameworks [33, 37, 39] and literature [18, 54–62]. This intervention aims to promote and improve cognitive factors, environmental support, and behavioral factors (Tables 1 and 2; Fig. 1) through a mobile self-management application, a self-management handbook, and professional support. Through self-management learning, we anticipate individual patients will acquire the knowledge, skills, and self-confidence to manage their own urinary incontinence and related occurrences. Moreover, we expect patients will learn to apply this knowledge to real-life situations and achieve goals set of the intervention.

**Fig. 2:**
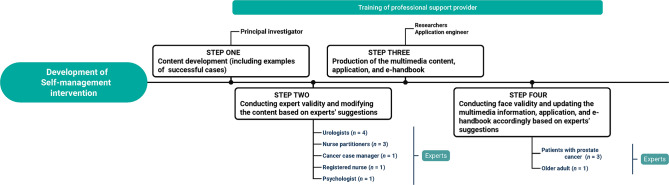
Steps used for the development of the self-management intervention

The developmental process for SMI-UI is illustrated in Fig. 2. The drafting stage involves developing multimedia content (Table 2; a new topic every two weeks for a total of six topics). The content was prepared by the principal investigator (PI) based on available literature and clinical experience. Following discussion with the research team, the content was reviewed by ten experts, including urologists, cancer case managers, nurse practitioners, psychologists, and registered nurses. The expert validity assessment with a 4-point scoring scale (1–4 points) for the six units revealed mean clarity scores ranging from 3.4 to 3.9 points and mean appropriateness scores ranging from 3.5 to 3.9 points. Content was modified based on suggestions by the experts, and thereafter multimedia films were produced. The multimedia content consists of images, animation, music, and sound. Once compiled, three patients with prostate cancer and one healthy older adult were invited to assess the initial version of the mobile self-management application, review the handbooks, view multimedia content, and offer feedback for refinement. Ultimately, revisions were made with a primary focus on improving the conciseness, clarity, and flow of the information presented in the mobile self-management application and multimedia content. The final layout and content of the handbooks received positive feedback from all four experts, and no further modifications were deemed necessary.

#### Mobile self-management application

**Fig. 3:**
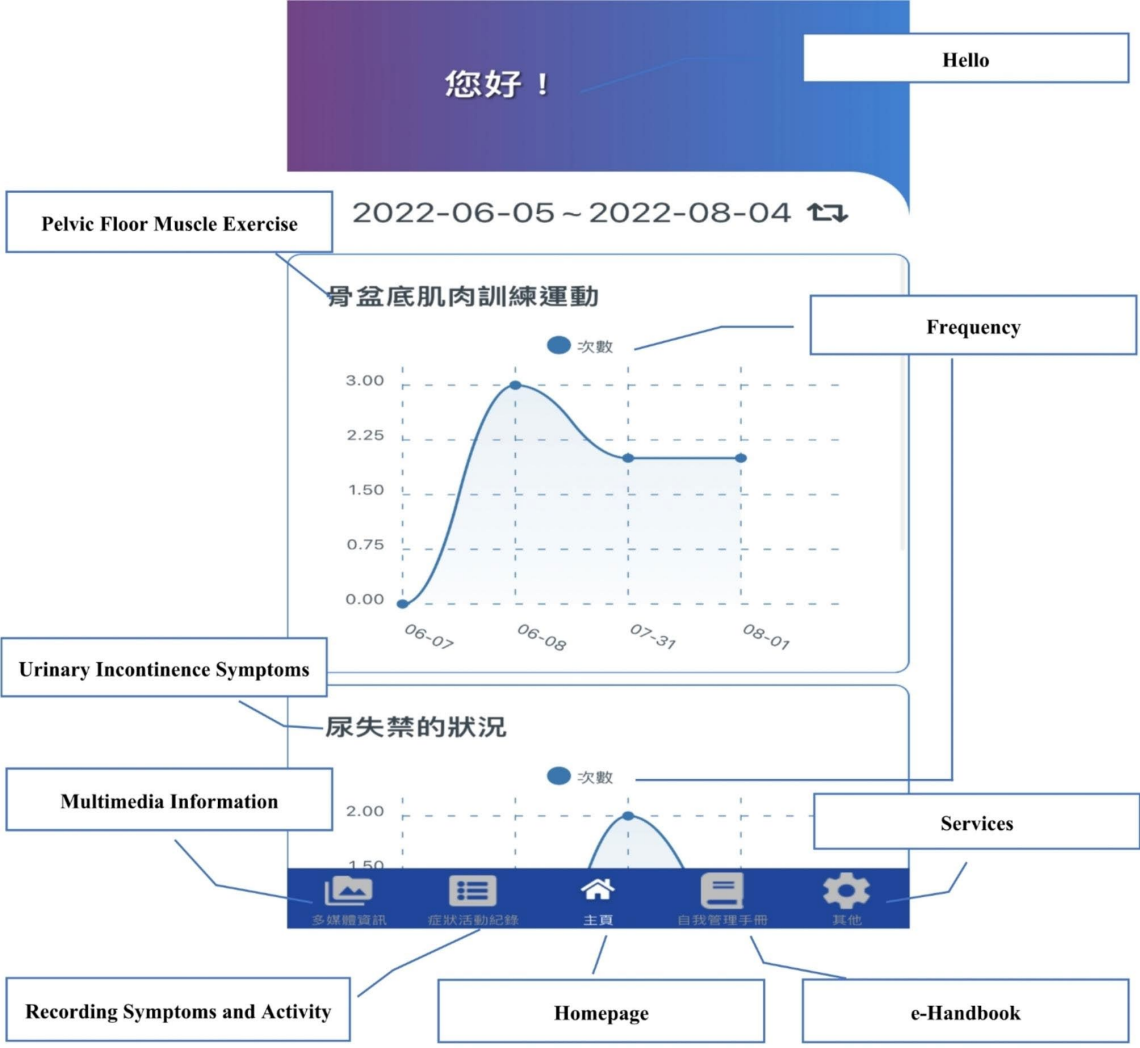
Self-management application

The mobile self-management application (Fig. 3) developed in this study can be installed on smartphones or tablet computers. This application incorporates numerous functions, including access to multimedia information and the self-management e-handbook; recording of urinary incontinence symptoms and converting the data into a line graph; recording of PFME status and diaper usage and converting the data into line graphs; providing immediate feedback; and receiving push notifications. The application also records the reading time of the multimedia information and the self-management e-handbook for each user. If fewer than four PFME instances are recorded, the application will inform the researcher via push notification, encouraging the user to continue to meet goals (Fig. 3).

The self-management e-handbook contains the following: (A) multimedia content corresponding to the application; (B) pages for symptom recording; (C) instructions for PFME; and (D) instructions for using the mobile self-management application.

#### Self-management handbook

The handbook is the hard copy of the self-management e-handbook.

#### Professional support

Telephonic professional support will be provided weekly by a trained nurse (professional support provider), whose primary tasks involve assessing the physical and mental conditions of each patient, evaluating PFME and urinary incontinence, ensuring patient self-care, explaining the use of the application, explaining and clarifying the provided information, discussing and assisting patients with goal-setting, and performing self-confidence assessments. When providing support through communication, professional support providers will maintain a pleasant atmosphere and focus on encouraging the patients. The professional support provider can share the experiences of other patients as required. On even weeks (week 2, 4, 6, 8, 10, 12), professional support will be scheduled at a time convenient for individual patients, and on odd weeks (week 1, 3, 5, 7, 9, 11), professional support will be scheduled at a convenient time after reviewing the information. Each session will last a minimum of 5–10 min and will be tailored to the individual patient’s needs as required. The professional support provider will record the results of each session at the session’s conclusion.

### Dietetic education information package

In addition to routine nursing support, individuals in the attention control group will receive a 20-min recording on a compact disc and a diet handbook for patients with prostate cancer, including an introduction, the principles of a well-balanced diet, and suggested dietary precautions. The handbook and multimedia video are a part of the multimedia psychosocial intervention developed in a previous study on the diet for patients with early prostate cancer [63].

### Routine nursing

In clinical settings, the routine nursing support for patients with urinary incontinence after radical prostatectomy includes guidance on the execution of PFME, assistance in learning through biofeedback if necessary, and the provision of extracorporeal magnetic stimulation treatment and oral medicine in accordance with the condition of each patient.

### Random assignment

Stratified and blocked random assignment will be utilized in this study. Three strata will be used: (A) 0–6 months, (B) 7–12 months, and (C) 13–24 months after surgery. A 2 × 2 block (block randomization) will be implemented at each level. Individual patients are to be assigned to the experimental and attention control groups at a ratio of 1:1. The random assignment code will be obtained by the principal investigator through a random number generator and will be placed in a sealed opaque envelope.

### Blinded design

The attention control group is to be used to maintain internal validity [41]. Clinical health-care providers will not be informed the group assignment of the patients in this study. Health-care providers, patients, and data collectors will be unaware of the study’s hypotheses and which group is the experimental group.

### Study procedure

**Fig. 4:**
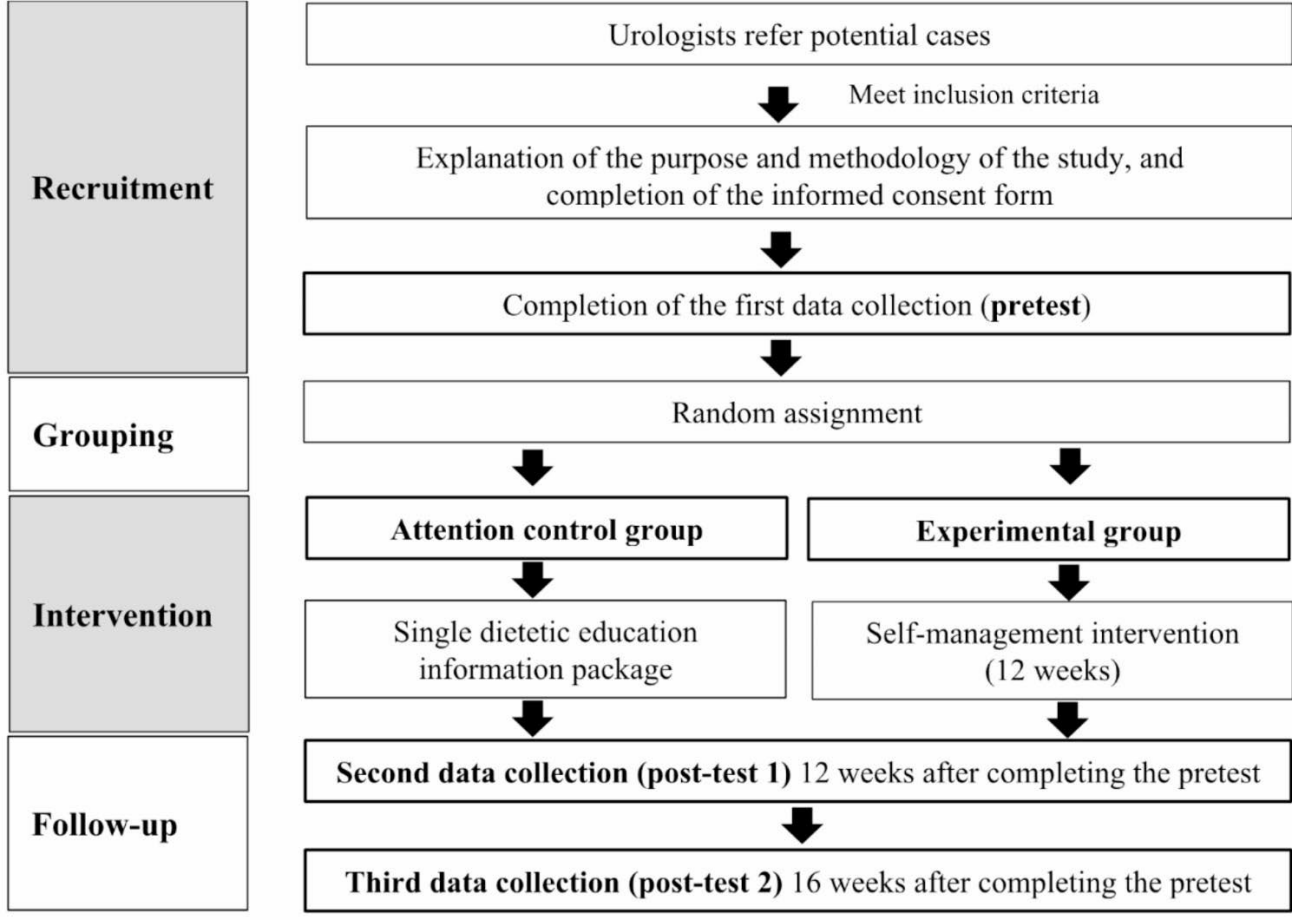
Flowchart of data collection

**Fig. 5:**
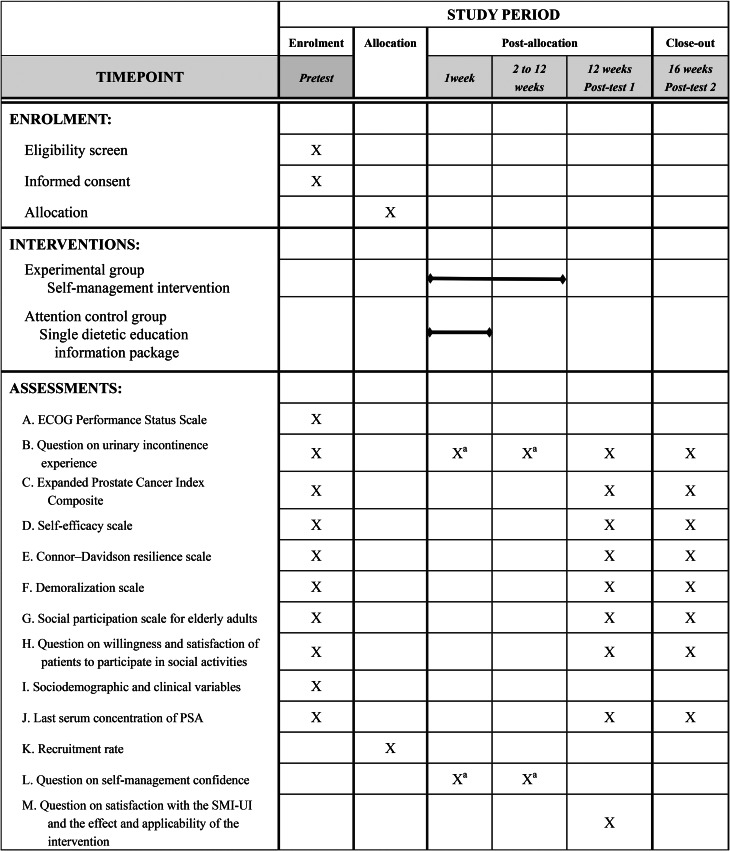
SPIRIT figure: Schedule of enrollment, interventions, and assessments. Note: ECOG = Eastern Cooperative Oncology Group, PSA = Prostate-specific antigen, SMI-UI = Self-management intervention for urinary incontinence, ^a^experimental group only

Figures 4 and 5 present an overview of the study procedure. The urologists will refer potentially suitable cases to the researchers. Subsequently, the researchers will determine whether these patients meet the inclusion criteria by reviewing their medical records and conducting interviews based on a question regarding urinary incontinence experience. The researcher will explain the purpose and process of the study to the patients who meet the inclusion criteria and invite them to participate in this investigation. After patients agree to participate and sign the consent form, they will complete the pretest questionnaire in a private area. Next, the researcher will open the random assignment envelope prepared by the principal investigator and assign the patients to either the experimental group or the attention control group. The experimental group will undergo a 12-week SMI-UI in addition to routine care, whereas the attention control group will receive routine care and a dietetic education information package. After posttests 1 and 2, data will be collected from the two groups at 12 and 16 weeks after the pretest, respectively. With the assistance of the researchers, the mobile self-management application will be installed on the smartphones or tablets of patients in the experimental group. In accordance with the steps for the operation of the application outlined in the self-management handbook, the patients will be taught to use the application. Thereafter, the researcher will provide each patient with SMI-UI activities for a duration of 12 weeks. Patients in the attention control group will be provided with a dietetic education information pack for home learning. Face-to-face post-test 1 and post-test 2 will be scheduled during patients’ follow-up visits. In a few cases where the timing of outpatient visits are outside of data collection hours, paper questionnaires will be mailed with the patient’s consent. As a token of appreciation for their contribution to the research, participants will receive a gift voucher worth NT$100 each time they complete a questionnaire.

### Management and monitoring of intervention fidelity

In this study, an Intervention Provider Handbook, a Case Interaction Record, and a research handbook will be developed. Explanatory meetings and training courses will be organized for the researchers to ensure that they clearly understand the study design, methods, processes, and relevant precautions. The SMI-UI will be performed by a trained nurse who will complete the required training before providing the intervention (intervention provider). In addition, the intervention provider will be required to complete the Case Interaction Record immediately after each session of professional support. The principal investigator will regularly review the interaction record form to assess the consistency and correctness of the provided intervention. The researcher will evaluate the time that individual patients spend reading the multimedia information and the e-handbook on the mobile self-management application and will invite patients to record the status of their urinary incontinence and the implementation frequency of PFME by using the application. The intervention provider will ask the patients some questions in each interaction to assess how they are learning about and implementing the intervention. The confidence of patients in applying new knowledge or skills to daily life will also be assessed through questions regarding self-management confidence (0–10 points). For those with a score of < 7 points, the unclear points will be clarified [18].

### Ethical considerations

This study has been approved by the institutional review board of the hospital recruiting individual patients (approval number: 201902235B0C505). The research plan will be registered before initiating the recruitment of the first research case (ClinicalTrials.gov ID: NCT05335967). The researchers will abide by the research ethics guidelines and maintain the privacy of patients (i.e., the identity of patients will be encoded). All patients will have the right to withdraw from the study at any time with no effect on their original treatment. The collection of research data is for academic purposes only.

### Data management and dissemination of results

The research data will be stored in the office of the principal investigator. The electronic files will be stored on a password-protected computer, and hard copies of the questionnaires and consent forms will be separately placed in opaque, locked cabinets. A researcher will input the questionnaire data into the IBM SPSS Statistics, and then two researchers will validate that the data have been entered correctly. Before the researchers perform statistical analysis, they will use descriptive statistics to understand the data distribution and confirm the absence of anomalous scores. The research results will be submitted to international peer-reviewed journals and conferences and provided to participants who are interested in them.

### Power analysis and sample size

Cohen’s *f* represents a standardized measure of the average effect [64] therefore, it is not affected by the varying score ranges of each scale. Based on the results of a previous study, the effect size f is 0.27 for the outcome variable of self-efficacy [65]. The sample size of this study was estimated using G*Power, with α = 0.05 and power = 80% according to repeated-measure analysis of variance (ANOVA) with two groups and three measurements. This study requires a sample size of 74 [66]. Based on this information and considering a study discontinuation rate of 17–18% [67], we plan to include a total of 86 patients (43 per group) in this study.

### Data analysis plan

All patient data will be included in the analysis. IBM SPSS Statistics (version 20.0; IBM Corp., Armonk, NY, USA) will be used to perform statistical analysis. Descriptive statistics (e.g., mean, standard deviation, median, frequency, and percentage) will be used to present data. The homogeneity of basic attributes, disease treatment variables, and outcome variables in the experimental and attention control groups at the pretest stage will be assessed through independent sample *t* tests and chi-squared tests. The effect of the intervention will be determined using a generalized estimating equation. Two-tailed tests will be used, and *P* values ≤ 0.05 will denote statistically significant differences.

## Discussion

This study is the first to investigate the effects of the developed SMI-UI on the urinary symptoms and disturbance, cancer-related self-efficacy, social participation and satisfaction, resilience, and demoralization of patients with prostate cancer. An application can improve self-care among patients with prostate cancer [17]. In this study, through the use of the mobile self-management application, health education handbook, and professional support, this intervention will guide patients through learning strategies for managing urinary incontinence. Moreover, patients will develop skills for the prevention and management of urinary incontinence as well as for the avoidance or reduction of the impact of urinary incontinence on daily life, social activities, and leisure activities. Patients can also record the severity of urinary incontinence, usage of diapers, and frequency of PFME implementation through the application, which is expected to help them implement self-management activities [17]. The application can also represent the data input of patients in iconic form and can provide immediate feedback, thereby helping patients with prostate cancer to strengthen their self-management of urinary incontinence.

Most patients and their families dislike staying in the hospital for a long time, especially during the COVID-19 pandemic [68]. This motivated our research team to develop the mobile self-management application, to design research-related activities that can be completed and understood within a limited time, and to provide telecare. This has led to the practice of professionals providing support through telephone calls with the help of a mobile self-management application and handbook. To improve the efficacy of telephonic communication, the topics scheduled for discussion in each unit after the introduction of successful cases are prepared in multimedia format, and sufficient time is allotted for patients to think and provide their feedback in the handbook. Thereafter, the intervention provider is to conduct individual telephone discussions with the patients to provide effective support.

This study began in May 2022, but because of the COVID-19 pandemic, many hospital beds had to be reassigned to patients with COVID-19, and nonemergency surgery and medical treatment had to be postponed or delayed in accordance with the national and hospital pandemic prevention policies. Therefore, the number of patients who underwent surgical treatment was considerably lower than that recorded before the pandemic [68]. The inclusion period for this study definitely therefore needs to be extended to reach the desired sample size to provide sufficient statistical power.

### Current study status

At the time of this submission, 68 of 90 participants have been recruited and randomized into two groups.

**Table 1:** Intervention strategies corresponding to key concepts of social cognitive theory

Variable		Intervention strategies					
		Self-management information in the application and handbook	PFME teaching and practice	Application functions: push notification, recording, and immediate feedback	Professional support	Encouragement and maintaining a harmonious atmosphere	Learning together with a family member
Individual cognitive factors	Self-efficacy	●^abcd^	●^a^		●^ac^	●^acd^	
	Outcome expectations	●			●		
	Knowledge	●	●				
Environmental factors	Observation learning	●			●		
	Social support			●	●		●
	Barriers and opportunities		●		●		●
Support behavioral factors	Behavioral skills	●	●	●	●		
	Intentions and goal setting	●			●		
	Reinforcement and punishment			●	●	●	●

*Note*: Strategies for improving self-efficacy

^a^performance accomplishment

^b^vicarious experience

^c^verbal persuasion

^d^emotional arousal

PFME = Pelvic floor muscle exercise

**Table 2:** Topics of self-management for urinary incontinence

Week	Topics	Outline of intervention content	Length of video (min)
1	Self-management - health guard	Postoperative urine incontinence mechanism, importance and objective of self-management, self-management guidelines	10:55
3	Rehabilitation – improve urinary incontinence	Pelvic floor muscle exercises teaching and practice, bladder training	13:56
5	Locked - non-incontinence when moving	Strategies and exercises for preventing urinary incontinence when moving (sneezing, laughing, lifting weights, exercising, abdominal exertion)	9:34
7	Build a new life - daily life	Skills for lowering the impact of leakage of urine on daily life	9:56
9	Build a new life - social activities	Precautions for participating in social and leisure activities	9:08
11	Establish a new goal - emotional management	Strategies for preventing negative emotions caused by urine incontinence	10:03

*Note*: All topics include experience-sharing by successful cases, case discussion, and self-confidence assessment

## Data Availability

No data was used for the research described in the article.
